# FLASH Radiotherapy: History and Future

**DOI:** 10.3389/fonc.2021.644400

**Published:** 2021-05-25

**Authors:** Binwei Lin, Feng Gao, Yiwei Yang, Dai Wu, Yu Zhang, Gang Feng, Tangzhi Dai, Xiaobo Du

**Affiliations:** ^1^ Department of Oncology, MianYang Central Hospital, Mianyang, China; ^2^ Institute of Nuclear Physics and Chemistry, China Academy of Engineering Physics, Mianyang, China; ^3^ Institute of Applied Electronics, China Academy of Engineering Physics, Mianyang, China

**Keywords:** FLASH radiotherapy, history, mechanisms, future, conventional dose-rate radiotherapy

## Abstract

The biological effects of radiation dose to organs at risk surrounding tumor target volumes are a major dose-limiting constraint in radiotherapy. This can mean that the tumor cannot be completely destroyed, and the efficacy of radiotherapy will be decreased. Thus, ways to reduce damage to healthy tissue has always been a topic of particular interest in radiotherapy research. Modern radiotherapy technologies such as helical tomotherapy (HT), intensity-modulated radiation therapy (IMRT), and proton radiotherapy can reduce radiation damage to healthy tissues. Recent outcomes of animal experiments show that FLASH radiotherapy (FLASH-RT) can reduce radiation-induced damage in healthy tissue without decreasing antitumor effectiveness. The very short radiotherapy time compared to that of conventional dose-rate radiotherapy is another advantage of FLASH-RT. The first human patient received FLASH-RT in Switzerland in 2018. FLASH-RT may become one of the main radiotherapy technologies in clinical applications in the future. We summarize the history of the development of FLASH-RT, its mechanisms, its influence on radiotherapy, and its future.

## Background

Radiotherapy is required by 60–70% of cancer patients during their treatment (1). Radiotherapy is the most widely used and effective anti-tumor therapy (2), but it can cause acute and late damage to healthy tissues. The dose delivered to the tumor is, hence, limited by the toxicity to nearby healthy tissue; this can mean that a tumor cannot be completely killed, and the efficacy of radiotherapy will be decreased. Thus, preventing or mitigating radiation-induced healthy tissue injury has always been a topic of particular interest in radiotherapy research. Modern radiotherapy technologies, such as intensity-modulated radiation therapy (IMRT), helical tomotherapy (HT), and proton radiotherapy can reduce radiation damage to healthy tissues. For example, IMRT reduces the incidence of grade 2–4 xerostomia in patients with head and neck cancers without compromising loco-regional control and overall survival (3). HT can also reduce the incidence of skin toxicity in breast cancer patients (4). The advantage of proton radiotherapy is that most of the energy is transferred to the position of the “Bragg peak,” and energy transfer outside of this point is very low (5). Thus, the radiation damage to healthy tissues outside the Bragg peak is relatively small, and proton therapy causes less radiation damage to healthy tissues than photon therapy when treating head and neck cancers, prostate cancer, and pediatric cancer, among others (6–8).

**Figure 1:**
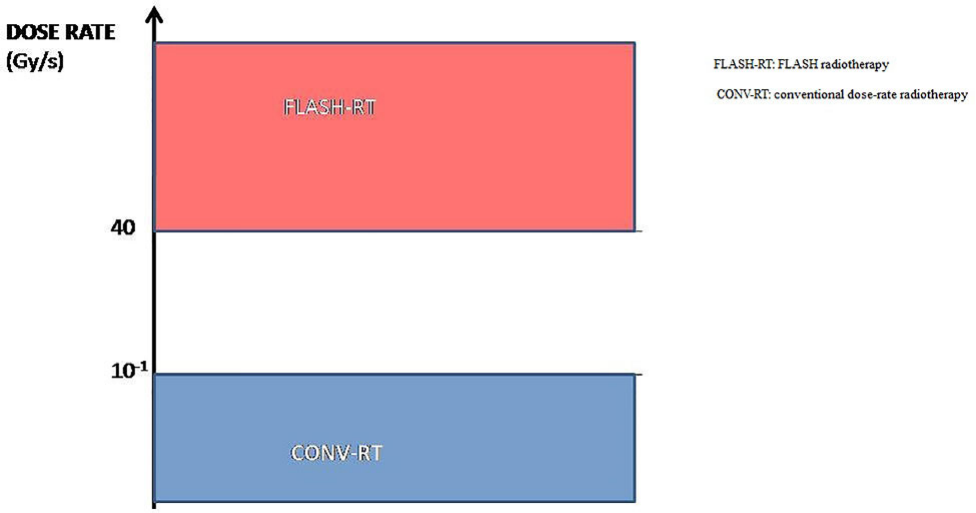
The dose-rate of FLASH-RT and conventional irradiation.

FLASH radiotherapy (FLASH-RT) is a novel radiotherapy technology defined as a single ultra-high dose-rate (≥ 40 Gy/s) radiotherapy. Compared with conventional dose-rate irradiation, FLASH irradiation is 400-fold more rapid than conventional irradiation (**Figure 1**). Recent animal experiments have shown that FLASH-RT can reduce radiation-induced damage in healthy tissues (9). In the first patient with T-cell cutaneous lymphoma who received FLASH-RT, the anti-tumor effect was rapid and long-lasting; moreover, only grade 1 epithelitis and grade 1 edema occurred in the soft tissues surrounding the tumor (10). In this first clinical use of FLASH-RT, the treatment time was only 90 ms. Compared with conventional dose-rate radiotherapy, the very short radiotherapy time is another advantage of FLASH-RT. Considering that FLASH-RT can reduce the damage to healthy tissue and the advantages of the short treatment time, we have reason to predict that FLASH radiotherapy may become one of the main radiotherapy technologies in clinical practice in the future.

In this paper, we summarize the history of development of FLASH-RT, its mechanism, its influence on radiotherapy, and its future.

## History of FLASH-RT

Before 2014, FLASH-RT was referred to as the flash effect, which was first reported by Dewey and Boag in 1959. Ultra-high dose-rate 1.5-MV X-rays were used to irradiate a bacterium, *Serratia marcescens* (11). This study showed that *S. marcescens* in a nitrogen-oxygen mixture containing 1% oxygen is more radiosensitive than when in 100% nitrogen with normal dose-rate irradiation (1000 rads/min). However, when the ultra-high dose-rate (10-20 kilorads/2 µs) was used, *S. marcescens* in the same nitrogen-oxygen mixture showed lower radiosensitivity, corresponding to anaerobic irradiation. In summary, Dewey and Boag’s study outlined that ultra-high dose-rate irradiation can protect bacteria when compared to conventional dose-rate irradiation. Similar results were observed in mammalian cells in later studies. Town reported that when mammalian cells received the same dose ultra-high dose-rate (3.5×10^9^ rad/s) irradiation, and the dose reached up to 1000 rads, one pulse had a higher survival rate than two pulses (12). Berry et al. showed similar results in hamster cells and HeLa cells using ultra-high dose-rate (1,000 rads for the 15-ns pulse) irradiation (13). A series of experiments showed that the flash effect is related to oxygen consumption (14–18). In 2014, Favaudon reported that using FLASH-RT to treat lung tumors can lead to a complete response and reduce the early and late toxicity affecting normal lung tissue; subsequently, FLASH-RT has become a topic of particular interest in radiation research (19). A series of studies have shown that FLASH-RT delivers dramatically reduced adverse side effects in the healthy tissue of mice (20–26), and this effect was greater in mini-pig and cat (27). Finally, a T-cell cutaneous lymphoma human patient received FLASH-RT, and long-lasting complete tumor response was achieved with fewer side effects than those expected with conventional radiotherapy (10). **Table 1** summarizes the preclinical and clinical evidence supporting FLASH-RT.

**Table 1:** Summary of preclinical and clinical evidence.

System	Author	Year	Irradiation		Modality ofradiation	models		Endpoint(s)	Main findings*	
			FLASH-RT	CONV-RT		Tumor	Normal tissue		Tumor	Normal tissue
Brain	Montay-Gruel P (26)	2020	12.5×103 -5.6×106 Gy/s	0.1Gy/s	electron	mice (glioblastoma)	_	tumor growth;cognitive function	similar antitumor effect	protective effect
	Montay-Gruel P (23)	2019	>100 Gy/s	0.07-0.1 Gy/s	electron	–	mice	cognitive function;ROS, neuronal structure, synaptic protein, neuroinflammation	–	fully preserved
	Simmons DA (24)	2019	200, 300Gy/s	0.13 Gy/s	electron	–	mice	cognitive function, neurodegeneration, neuroinflammation	–	protective effect
	Montay-Gruel P (21)	2018	37 Gy/s	0.05 Gy/s	X-ray	–	mice	cognitive function, Cell proliferation, GFAP	–	protective effect
	Montay-Gruel P (20)	2016	0.1,1, 3, 10, 30, 100,500 Gy/s, 5.6 MGy/s		electron	–	mice	cognitive function	–	protective effect above 30 Gy/s, fully preserved above 100 Gy/s
Intestine	Venkatesulu BP (28)	2019	35Gy/s	0.1 Gy/s	electron	–	mice	toxicity, survival	–	No protection effect
	Billy W. Loo (9)	2017	210 Gy/s	0.05 Gy/s	electron	–	mice	survival	–	protective effect
Lung	Fouillade C (29)	2020	40–60GY/S	?	electron	–	mice	cell proliferation, DNA damage, inflammatory genes		protective effect
	Buonanno M (22)	2018	0.025 Gy/s - 1500 Gy/s		proton	–	human lung fibroblasts	cell survival, b-gal, TGFb		protective effect
	Favaudona V (30)	2015	>40 Gy/s,	< 0.03Gy/s	electron	mice(lung tumor)	mice	tumor growth, apoptosis, lung fibrosis	similar antitumor effect	protective effect
	Favaudon V (19)	2014	≥40 Gy/s	< 0.03Gy/s	electron	mice(lung tumor)	mice	tumor growth, early and late complications	similar antitumor effect	protective effect
Skin	Bourhis J (10)	2019	166.7Gy/s	–	electron	patient(lymphoma)	–	tumor response; Soft tissue toxicity	complete response	grade 1 epithelitis, grade 1 oedema
	Vozenin MC (27)	2018	300 Gy/s	0.083 Gy/s	electron	cat(squamous carcinoma	pig	skin toxicity, PFS	PFS at 16 months was 84%	protective effect
Blood	Chabi S (25)	2020	200Gy/S	<0.072 Gy/S	electron	mice(leukemia)	mice	tumor growth, normal hematopoiesis	similar antitumor effect	protective effect
Other	Adrian G (31)	2020	600 Gy/s	0.233 Gy/s	electron	prostate cancer cells	–	survival	flash effect depends on oxygen concentration	
	Beyreuther E (32)	2019	100 Gy/s	0.083 Gy/s	proton	–	zebrafish embryo	survival	–	Similar toxicity except for pericardial edema at one dose point(23Gy)

FLASH-RT, FLASH radiotherapy; CONV-RT, conventional dose-rate radiotherapy; *Effects of FLASH-RT compared with CONV-RT.

## Mechanism of FLASH-RT

**Figure 2:**
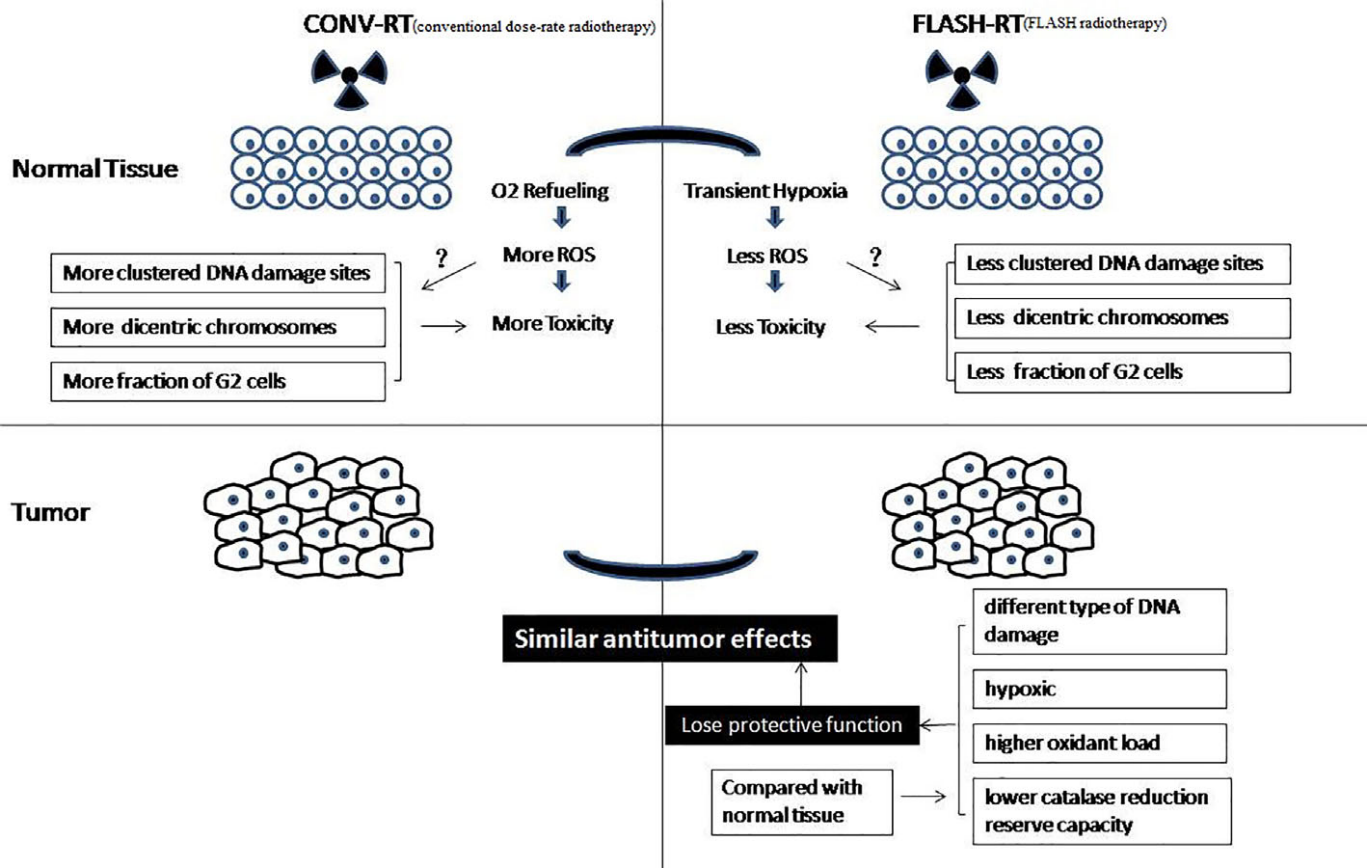
Biological mechanism of FLASH-RT.

The biological mechanism of FLASH-RT is very complex (**Figure 2**). Dewey and Boag first reported the effects of ultra-high dose-rate, which showed that hypoxia was induced following high dose-rate radiotherapy of bacteria (11). This phenomenon can be explained by local oxygen consumption because the rapid deposition of radiation energy occurs too fast to maintain sufficient oxygenation levels. A series of studies have shown that ultra-high dose-rate irradiation can induce the protection of mammalian cells through transient hypoxia (including cancer cells) (14–18). A recent study showed that hyperoxia can eliminate flash effects in mice (33). Another theory is that the number of DNA damage sites in FLASH-RT is less than that following conventional dose-rate irradiation. Some authors have shown that FLASH-RT causes fewer dicentric chromosomes to be formed than conventional dose-rate irradiation, and there was a difference in G2 cell cycle arrest after FLASH-RT and conventional dose-rate irradiation (34–37). Another study showed that radiation with short pulses leads to fewer late side effects in healthy tissue than conventional dose-rate irradiation (38). Recently, Kim et al. showed that myosin light chain activation plays an important role in the separation of biological effects between FLASH-RT and conventional dose-rate irradiation (39).

However, those studies did not demonstrate the same protective effect between healthy and tumor tissues *in vivo*. In vivo, FLASH-RT leads to differential responses between healthy and tumor tissues. Compared with conventional dose-rate irradiation, FLASH-RT can reduce radiation-induced lung fibrosis and has the same antitumor effectiveness in mice (19). Why are there differential responses between healthy and tumor tissues *in vivo*? A theory is that the different types of DNA damage caused by FLASH-RT and conventional dose-rate irradiation result in the differential responses of healthy and tumor tissue (33, 40, 41). Another theory is that solid tumors are hypoxic, so they will not be protected from the transient hypoxia caused by FLASH-RT, while healthy tissues will be, resulting in the differential effect (23, 33). In addition, some authors believe that cancer cells and normal cells have different abilities to scavenge hydrogen peroxide products. FLASH-RT instantaneously consumes oxygen in all local tissues and produces hydrogen peroxide products. Healthy tissue cells have a lower oxidant load and higher catalase reduction reserve capacity; therefore, healthy tissue can remove hydrogen peroxide products more easily than tumor tissue (42).

In general, previous studies have agreed that FLASH-RT leads to local oxygen consumption that is much faster than tissue oxygenation, which results in transient radiation-induced hypoxia. Interestingly, some researchers believed that FLASH-RT using carbon ions would improve therapeutic ratio with greater toxicity in the tumor due to the generation of oxygen at the spread-out Bragg peak (43). Hence, the mechanism behind the differential responses of healthy and tumor tissues remains unclear and the various explanatory hypotheses require more experimental verification (44).

## Influence on Radiotherapy

FLASH-RT might potentially change the theories of radiobiology as follows. The first change may be in the five Rs of radiobiology: DNA repair, reoxygenation, repopulation, redistribution, and intrinsic radiosensitivity (45). The delivery time of FLASH-RT is too short for reoxygenation, repopulation, and redistribution to occur, or reoxygenation, repopulation, and redistribution may occur but cannot influence the effect of radiotherapy because FLASH-RT is performed only once. Therefore, FLASH-RT may be related to two Rs: DNA repair and intrinsic radiosensitivity. The second change may be the limit dose of healthy tissue because preclinical studies have confirmed that compared with conventional dose-rate irradiation, FLASH-RT needs a higher dose to cause the same degree of toxicity. As a result, when healthy tissue is irradiated in FLASH-RT, its α/β value will change. Conventional dose-rate irradiation (15 Gy) triggers lung fibrosis, but higher dose FLASH-RT (20 Gy) does not cause lung fibrosis after 36 weeks. Regarding skin toxicity, both 20 Gy and 15 Gy FLASH-RT do not exhibit macroscopic signs of cutaneous lesions, but 17-Gy conventional dose-rate irradiation can lead to severe cutaneous lesions within the irradiated field (19). Compared with conventional dose-rate radiotherapy, FLASH-RT also shows protective effects in other healthy tissues, including the brain and digestive tract (9, 16, 20, 21, 27, 46, 47). A series of animal experiments have shown that the tolerance of healthy tissues is increased; thus, when FLASH-RT is used, the α/β value of healthy tissues will differ from the current value using in conventional dose-rate irradiation. The third potential change is the comprehensive treatment strategy. For example, how can radiotherapy be combined with chemotherapy? Because FLASH-RT is performed only once for a very short time, concurrent chemoradiotherapy is not possible; only neoadjuvant and adjuvant chemotherapy can be administered. The fourth change may be the fraction of radiotherapy. If FLASH-RT technology is used in clinical practice and gradually develops, single fraction radiotherapy will be widely used to replace the current multiple fractions of radiotherapy.

## The Future of FLASH-RT

Additional animal experiments are needed to prove that FLASH-RT can provide more protection to healthy tissues. Favaudon et al. reported that FLASH-RT has lower rates of acute and late pulmonary toxicity after thoracic irradiation of mice than conventional dose-rate irradiation at the same dose (19). However, the two groups were irradiated with different rays (FLASH-RT with 4.5 MeV electrons *vs*. conventional dose-rate irradiation with Cs-137 photons). The protective effect of FLASH-RT in Favaudon′s study may be due to the different physical characteristics of electrons and photons. Because the energy of 4.5 MeV of electrons is mainly deposited on the surface, FLASH-RT delivers a lower dose to deep tissue (lung) when compared with that with conventional dose-rate irradiation. In another study of the brains of mice, FLASH-RT was applied using X-ray (21). In this study, the shape of the radiation field was different, although the circular radiation field of FLASH-RT was equivalent in area to the square radiation field of conventional dose-rate radiotherapy. Future experiments should be designed to use the same conditions, for example, by using the same rays and same shape of radiation field. Moreover, other organs should also be examined in future experiments.

Different modified irradiation systems can produce FLASH dose-rates, including electron linear accelerators, synchrotron light sources, and proton accelerators. Electron beam radiotherapy could be a source of FLASH-RT. The two prototype linear accelerators were modified to generate 4.5 MeV and 6 MeV electron beams with dose-rates in the thousands of Gy/s (19). High dose-rate (18000 Gy/s) X-ray from a synchrotron light source can also produce a FLASH effect (21). The proton cyclotron can also produce high-energy rays with a dose-rate of more than 40 Gy/s for FLASH-RT (48). However, most of the equipment can only be used in animal experiments. Only one setup was used to treat a T-cell cutaneous lymphoma patient, and the radiotherapeutic fields of this setup were small. In the future, equipment should be modified to allow radiotherapy over larger fields that are suitable for treating cancer patients. Electrons, X-rays, and protons can be used for FLASH-RT; however, the electron beam is only suitable for superficial tumors. Protons are suitable for the treatment of deep tumors. The first proton FLASH-RT equipment has obtained investigational device exemption (IDE) for clinical trials from the US Food and Drug Administration (49). However, the equipment for proton FLASH-RT is expensive. Because X-ray equipment is widely used all over the world and cheaper than that required for proton therapy, it is also suitable for treating deep tumors. Therefore, in our opinion, future development in the equipment for FLASH-RT should be focused on modifying X-ray equipment.

Future FLASH-RT devices may need to have the function of multiple-field conformal radiation. Multiple-field conformal radiation can reduce healthy tissue toxicity compared to the use of a single regular field or two regular fields. Multiple-field conformal radiation can be achieved using mechanical gantry rotation and movement of the multileaf collimator. In a previous study, one-pulse FLASH-RT provided greater protection than two-pulse therapy at the same dose and dose-rate, so one pulse will be the best choice (13). The time for one pulse is too short for the movement of the multileaf collimator and mechanical gantry rotation, so a multi-mechanical gantry will be needed, and the multileaf collimator should be moved to the corresponding position in advance to form a conformal region. Equipment similar to the Gamma Knife can meet the above requirements, but it will be very different from the Gamma Knife. Based on the above principles, some researchers have developed a PHASER radiotherapy system that can provide highly conformal intensity modulated FLASH-RT for tumors (50). However, there is still a long way to go before it can be used clinically.

Furthermore, before FLASH-RT is used clinically, two problems need to be solved. First, because of the differences between animal models and humans, the FLASH effect should be confirmed in cancer patients. Acute and late toxicity in different organs should be monitored. Second, because FLASH-RT can be completed in a single sitting, the definitive irradiation dose for different cancers needs to be redefined. Radiation oncologists should rebalance the effect of irradiation and healthy tissue toxicity and then define the radical irradiation dose. This may require the treatment of many cancer patients and a long time before this is satisfactorily defined. It may take many years before FLASH-RT becomes a mainstay radiotherapy technology in clinical applications.

## Conclusion

From 1959 to 2020, FLASH-RT has progressed from bench to bedside. FLASH-RT causes rapid depletion of oxygen, eliciting transient hypoxia. However, the exact mechanism of differential responses between healthy and tumor tissues is unclear. FLASH-RT may bring changes in radiobiology, limit radiation doses to healthy tissue, and promote new methods of combining radiotherapy with other antitumor treatments. Before FLASH-RT becomes the main radiotherapy technology in clinical application, more animal experiments are needed. The delivery should be modified to fit larger fields of radiotherapy, involve conformal radiation to multiple fields, redefine the dose limits to healthy tissue, and radical irradiation dose of cancer. In conclusion, for FLASH-RT, the road is tortuous but the future is bright.

## Author Contributions

BL and FG draft the manuscript, YY, DW, YZ, GF, and TD participated in the data review and collection for the study. XD conceived of the study, and participated in its design and coordination. All authors contributed to the article and approved the submitted version.

## Conflict of Interest

The authors declare that the research was conducted in the absence of any commercial or financial relationships that could be construed as a potential conflict of interest.
